# Curcumin preserves cognitive function and improve serum HDL in chronic cerebral hypoperfusion aging-rats

**DOI:** 10.1186/1750-1326-7-S1-S3

**Published:** 2012-02-07

**Authors:** Mingyuan Tian, Linhui Wang, Gang Yu, Bin Liu, Yu Li

**Affiliations:** 1 Department of Pathology, Chongqing Medical University, Chongqing 400016, China; 2 Institute of Neuroscience, Chongqing Medical University, Chongqing 400016, China; 3 Chongqing Key Laboratory of Neurobiology, Chongqing Medical University, Chongqing 400016, China; 4 Department of Neurology, The First Affiliated Hospital, Chongqing Medical University, Chongqing 400016, China

## Background

Chronic cerebral hypoperfusion is believed to be a critical factor on the occurrence of AD and VaD for which there is no effective therapy currently. It has been reported that high levels of circulating high density lipoprotein (HDL) can reduce the risk of cardiovascular disease. Although the relationship between cognitive function and serum lipids has captured attention, previous studies have shown ambiguous results. We investigated the effects of Curcumin on cognitive impairment and serum HDL levels and investigated whether increasing serum HDL levels ameliorate VAD-like cognitive deficits in rats induced by permanent occlusion of bilateral common carotid arteries (2VO).

## Method

Male Sprague-Dawley rats were subjected to permanent occlusion of bilateral common carotid arteries (2VO) to produce chronic cerebral ischemia. Animals were randomly divided into 5 groups: normal control group, sham-operated group, 2VO group, 2VO+Curcumin100mg/kg group, 2VO+ Curcumin50mg/kg group. Low doses of Curcumin (50mg/kg) or high doses of Curcumin (100mg/kg) were dissolved in DMSO. All animals were injected intraperitoneally with DMSO solution of Curcumin or a same volume of normal DMSO after surgery. Each group was injected once daily for four consecutive weeks. The spatial learning capacity and cognitive function of these animals was assessed in the Morris water maze 30 days after the onset of 2VO. Serum concentrations of high-density lipoprotein (HDL) were measured. Associations between serum concentrations of HDL and cognitive function were investigated for each group.

## Result

Morris water maze for spatial orientation abilities showed that rats in 2VO group develop impaired learning and cognitive deficits, injection intraperitoneally of Curcumin can attenuate cognitive impairment ( P<0.05). 2VO+Curcumin group had higher serum HDL cholesterol levels than that of 2VO group (P<0.05). No significant differences were found in the serum level of HDL among 2VO+Curcumin group rats, sham-operated group rats and normal control group rats. Further more, plasma HDL levels correlated significantly with cognitive function.

## Conclusion

Our findings suggest that Curcumin have a protective influence on cognitive function and apparently increase serum HDL in 2VO group rats. Furthermore, serum HDL levels have positive association with cognitive function. The relation between Curcumin and HDL is hoped for new preventive and therapeutic strategies for cognitive deficits.

